# Psychiatric morbidity and work participation in patients with congenital ventricular septal defects: a case-controlled study

**DOI:** 10.1093/ehjqcco/qcad072

**Published:** 2024-01-04

**Authors:** Filip Eckerström, Vibeke Elisabeth Hjortdal, Charlotte Ulrikka Rask, Camilla Nyboe

**Affiliations:** Department of Cardiothoracic Surgery, Copenhagen University Hospital, Blegdamsvej 9, DK-2100 Copenhagen, Denmark; Department of Clinical Medicine, Copenhagen University, Blegdamsvej 9, DK-2100 Copenhagen, Denmark; Adult Congenital Heart Disease Unit, Department of Medicine, Sahlgrenska University Hospital, Diagnosvägen 11, SE-416 85 Gothenburg, Sweden; Department of Molecular and Clinical Medicine, Institute of Medicine, Sahlgrenska Academy, University of Gothenburg, Medicinaregatan 3, SE-405 30 Gothenburg, Sweden; Department of Cardiothoracic Surgery, Copenhagen University Hospital, Blegdamsvej 9, DK-2100 Copenhagen, Denmark; Department of Clinical Medicine, Copenhagen University, Blegdamsvej 9, DK-2100 Copenhagen, Denmark; Centre for Child and Adolescent Psychiatry, Aarhus University Hospital Psychiatry, Palle Juul-Jensen Boulevard 99, DK-8200 Aarhus, Denmark; Department of Clinical Medicine, Aarhus University, Palle Juul-Jensen Boulevard 99, DK-8200 Aarhus, Denmark; Department of Clinical Medicine, Aarhus University, Palle Juul-Jensen Boulevard 99, DK-8200 Aarhus, Denmark; Department of Cardiothoracic and Vascular Surgery, Aarhus University Hospital, Palle Juul-Jensen Boulevard 99, DK-8200 Aarhus, Denmark

**Keywords:** Congenital heart disease, Ventricular septal defect, Psychiatric disorders, Work participation, Education, Nationwide

## Abstract

**Background:**

The burden of psychiatric morbidity, level of education, and work participation are currently unknown in patients with congenital ventricular septal defects (VSD).

**Methods and results:**

In a Danish population-based cohort study using nationwide medical registries, the burden of psychiatric disorders, use of psychotropic agents, level of education, and work participation were examined in patients with isolated congenital VSD and controls from the general population matched by age and sex. Subjects with known chromosomal abnormalities were excluded. To compute estimates, Cox proportional regression model, Fine and Gray's competing risk regression, and Kaplan–Meier failure function were used. We included 8006 patients and 79 568 controls born before 2018. Median follow-up was 23 years. Compared with controls, patients with VSD displayed a hazard ratio (HR) of 1.24 [95% confidence interval (CI): 1.17–1.32] for any psychiatric disorder where the hazard for intellectual disabilities was most pronounced [HR of 3.66 (95% CI: 2.98–4.50)]. The use of psychotropic agents was higher in patients compared with controls [HR 1.14 (95% CI: 1.09–1.20)]. The work participation was lower in patients with VSD compared with controls (*P* < 0.001) and was lower in patients with VSD with a psychiatric disorder compared with those without (*P* < 0.001). The 40-year cumulative incidence of permanent social security benefits was 29% in patients with psychiatric disorders (vs. 21% in controls with psychiatric disorders) and 8% in patients without psychiatric disorders (vs. 4% in controls).

**Conclusion:**

Patients with isolated VSD suffer from a higher burden of psychiatric disorders and display lower work participation compared with matched controls from the general Danish population. It is important to consider longer-term impacts on mental health, education, and subsequent employment in patients with VSD, in addition to cardiovascular effects, as these factors severely affect quality of life and have direct socioeconomic implications on an individual and societal level.

## Graphical Abstract

**Graphical Abstract:**
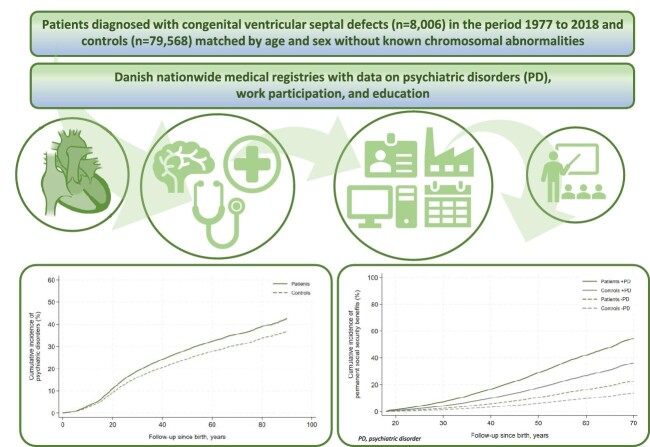

Key learning points **What is already known**:

- Higher rates of unemployment, disease-related work absences, and limitations to work have been reported in adults with unselected congenital heart diseases compared with the general population.
- Patients with congenital ventricular septal defects (VSD), both small unrepaired and larger surgically closed defects, carry a higher burden of mortality and cardiovascular morbidity and display significant early structural cerebral abnormalities, impaired neurodevelopment, and dysfunctional cognition abilities compared with the general population.
- Chronic somatic and psychiatric morbidity has a well-known negative impact on work participation; thus, patients with VSD could be at risk for an increased rate of unemployment and permanent social security benefits.

**What the study adds**:

- Work participation was lower among patients with VSD compared with controls from the general population where 13% of the patients and 7% of the controls received permanent social security benefits at the age of 40 years.
- Psychiatric morbidity was more common in patients with VSD compared with controls and was associated with a higher likelihood of retrieving permanent social security benefits.
- The increased burden of psychiatric morbidity could possibly be explained by yet unknown congenital extracardiac abnormalities. The longer-term outcome for patients with isolated congenital VSD is far from uncomplicated, and it is crucial to take a broader perspective in the longitudinal follow-up of children and adults with congenital heart diseases focusing not only on cardiovascular morbidity but also on mental health, education, and subsequent employment.

## Introduction

The adult population of patients with congenital heart disease is today greater than that of children,^1,2^ which is ultimately a result of improved and successful patient-tailored treatment. Consequently, attention has been drawn towards quality of life and prevention, detection, and treatment of morbidity. Higher rates of unemployment, disease-related work absences, and limitations to work have been reported in adults with unselected congenital heart diseases compared with the general population.^3–5^ Furthermore, from a Danish nationwide cohort study of 2277 patients, we know that isolated atrial septal defect (ASD) is associated with an increased burden of somatic^6–8^ and psychiatric^9,10^ morbidity and lower work participation^11^ compared with the general Danish population. Whether patients with ventricular septal defect (VSD) demonstrate similar pattern is unknown. We recently elucidated a lower than anticipated survival^12^ and a substantial burden of somatic morbidity^13^ in patients with isolated congenital VSD,^13^ the most common congenital cardiac malformation. Chronic somatic morbidity has a well-known negative impact on work participation; thus, patients with VSD could be at risk for an increased rate of unemployment and permanent social security benefits. Additionally, significant early cerebral abnormalities,^14^ impaired neurodevelopment,^15^ and dysfunctional cognition abilities^16^ have recently been demonstrated in patients with VSD suggesting that long-standing mental health issues might burden this population. Using a nationwide cohort of patients with isolated VSD we aimed to investigate the burden of psychiatric morbidity, work participation, and level of education in comparison with the general Danish population.

## Methods

### Ethics

The Danish Data Protection Agency (j. nr. 1–16-02–184-19) approved the study. Pseudo anonymous data were delivered from Statistics Denmark and informed consent from included patients and controls was therefore not required. In accordance with the directive from the central authority of Statistics Denmark, the data underlying this article cannot be shared publicly.

### Study population and design

All citizens in Denmark have access to public healthcare, free of charge, irrespective of disease, and irrespective of category of healthcare. Every healthcare contact and associated hospital data are registered and linked to the unique personal identification number provided for all the citizens since 1968. The linkage between the personal identification number and the hospital data generates a network of nationwide medical registries available for research. The Danish National Patient Registry (DNPR)^17^ was used to identify patients diagnosed with VSD in the period 1977 to 2018. Patients were identified by codes according to the 8th and 10th editions of the International Classification of Disease (ICD) (746.39 and Q21.0, respectively). Concomitant congenital cardiac malformations were similarly identified, except for bicuspid aortic valve; see Supplementary material online, *Table S1* for specific codes. VSD closure procedures were identified using the procedure codes specified in Supplementary material online, *Table S2*. Ten controls per case matched by birth year and sex were identified using the Danish Civil Registration System.^17^ Controls were sampled so that they did not have a congenital cardiac malformation and were alive at the time of VSD diagnosis of their matched case. The structure and the content of the nationwide registries as well as how they have been utilized in population-based research have been thoroughly described elsewhere.^12,13^

### Psychiatric morbidity and prescription of psychotropic agents

All inpatient hospital contacts with a psychiatric diagnosis from 1970 to 2018 were identified in the Danish Central Psychiatry Registry. Outpatient hospital contacts were added from 1995. Psychiatric morbidity was defined as psychiatric diagnoses according to the 8th and 10th editions of the ICD. The codes in the range F10 to F98 were used and are presented in detail in the Supplementary material online, *Table S3*.

The Danish National Prescription Registry was used to identify use of psychotropic agents. The registry contains all prescription drugs redeemed from Danish community pharmacies since 1994 with individual level information on the Anatomical Therapeutic Codes on prescriptions dispensed to all individuals including residents at care facilities. Redeemed prescriptions for psychiatric medicine defined as antipsychotic medicine, antidepressants, anxiolytics, hypnotics, and psychological stimulants were identified. In Denmark a prescription is obligate to obtain these psychotropic agents. Specific codes are outline in Supplementary material online, *Table S4*.

### Work participation and education

In Denmark social security benefits and services are tax funded and available to all citizens in need, irrespective of affiliation to the labour market. Data on work participation was retrieved from the DREAM database, which contains information on all paid social security benefits registered on a weekly basis from 1991 to 2018.^18^ Short-term sick leave (1–30 days) is paid by the employer, in the case the citizen is employed. Short-term sick leave for unemployed citizens is paid by the municipality. Long-term sick leave (>30 days) is paid by the municipality. The database covers benefits paid by the municipality to the employee and self-employed citizens but does not cover short-term sick leave paid by the employer. The database covers benefits paid to the employees and self-employed citizens but does not cover short-term sick leave paid by the employer. Using the DREAM database, following categories were created: (i) work (including maternity leave and education), (ii) unemployment (all citizens including both those with and without private insurance), (iii) long-term sick leave (paid by the municipality), and (iv) permanent social security benefit, including disability pension and flexi-job (a job created for persons with limited and permanently reduced working capacity). The Danish National Education Registry was used to retrieve data on the highest level of completed education since 1981.

### Statistics

Patients and controls were followed from birth (index date) until death or end of follow-up (31 December 2018), whichever came first. All controls were alive at the time of the VSD diagnosis of their matched case.

Medians with interquartile range (IQR) was used to describe descriptive continuous data. Dichotomous data were presented as absolute numbers with percentage of total numbers.

Cumulative incidence of psychiatric disorders and use of psychotropic agents were computed using Fine and Gray's regression model with death as competing risk. Cox proportional regression model was used to compute hazard ratios (HRs) with a 95% confidence interval (CI) for first contact (in- or out-patient hospital contacts) with a psychiatric disorder diagnosis with risk beginning at birth and underlying timescale being years since birth. Patients with VSD were compared with matched controls. Use of psychotropic agents in patients with VSD was analysed in the time period 1994 to 2018 using Fine and Gray's regression model with death as competing risk and cox proportional hazard regression model for first redeemed prescription for psychiatric medicine since VSD diagnosis compared with matched controls. Risk began at birth and underlying timescale was years since birth.

For each patient and matched controls, the total number of weeks from either the age of 18 years or the start of the DREAM database up until the end of follow-up, death, or retirement (not disability retirement) was generated. The proportion of weeks in the four categories defined previously (work, unemployment, long-term sick leave, and permanent social security benefit) was calculated. Fisher's exact test was used to compare VSD patients with matched controls. The date at which the first permanent social security benefits was initiated, was identified and used in a Cox proportional regression model comparing VSD patients with their matched controls, starting at the age of 18 years, using time since birth as underlying timescale. In order to account for competing risk from mortality and determine the cumulative incidence of permanent social security benefits in VSD patients and the matched controls, Fine and Gray's competing risk regression was utilized. Kaplan–Meier failure function was used to compute cumulative mortality and cumulative incidence of permanent social security benefits. Log-minus-log plots were used to graphically verify the assumption of proportional hazards. Statistical tests were two-tailed and *P*-values<0.05 considered significant. All statistical analyses were performed using STATA 17 (StataCorp. LP, College Station, TX, USA).

## Results

### Study population

**Figure 1:**
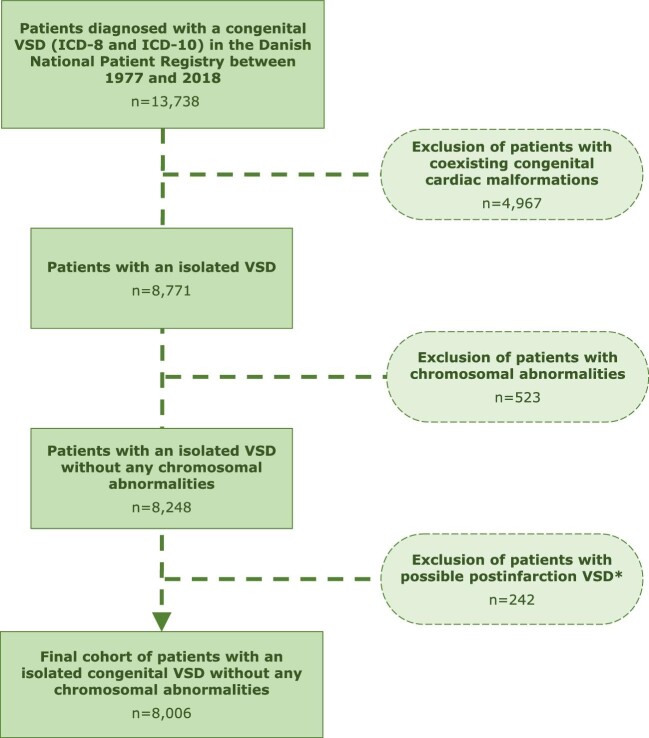
Flowchart of the inclusion process of patients with an isolated congenital ventricular septal defect.

The patient cohort consisted of 8006 patients with an isolated congenital VSD after exclusion of patients with coexisting congenital cardiac malformations (*n* = 4967) and patients with known chromosomal abnormalities (*n* = 523). The control cohort consisted of 79 568 matched controls without known chromosomal abnormalities from the general Danish population. Median follow-up was 22.8 years (11.5–37.2) and the majority (58%) of the patients were diagnosed within the first year of life. *Figure 1* graphically illustrates the formation of the study population. Demographics of the study population are presented in *Table 1*. VSD closure was performed on approximately 10% of the cohort, predominantly by surgery (*n* = 674) before the age of 6 years. During follow-up, 518 (6.4%) patients (465 unrepaired and 53 surgically closed) and 2475 (3.1%) controls died, depicted in Supplementary material online, *Figure S1*.

**Table 1:** Demographics

	Patients (*n* = 8006)	Controls (*n* = 79 568)	*P*-value
Female sex, *n*	4123 (51.4)	40 961 (51.4)	
Follow-up, *years*	22.8 (11.5–37.2)	23.6 (12.2–38.1)	
Age at VSD diagnosis, *years*	0.5 (<0.1–7.4)	*NA*	
VSD closure, *n*	628 (8.5)	*NA*	
Age at VSD closure, *years*	1.4 (0.5–5.7)	*NA*	
Pulmonary hypertension, *n*	70 (0.9)	61 (<0.1)	
Highest education^†^	4556 (56.9)	47 328 (59.5)	
Basic	1233 (27.1)	10 642 (22.5)	<0.0001
Youth	793 (17.4)	8257 (17.5)	0.9674
Vocational	1233 (27.1)	13 218 (27.9)	0.2193
Higher	1297 (28.5)	15 211 (32.1)	<0.0001
Short cycle	198 (3.6)	2076 (3.7)	0.9099
Medium cycle	709 (12.9)	8084 (14.2)	0.0083
Long cycle	363 (6.6)	4738 (8.4)	<0.0001
PhD and other	26 (0.5)	301 (0.5)	0.6950

Data are presented as absolute numbers with percentage of total numbers or as medians with IQR (*P*_25_–*P*_75_) in patients with isolated congenital ventricular septal defect and matched controls from the general Danish population. ^†^Proportion calculated from patients alive at 18 years.

### Psychiatric morbidity

**Figure 2:**
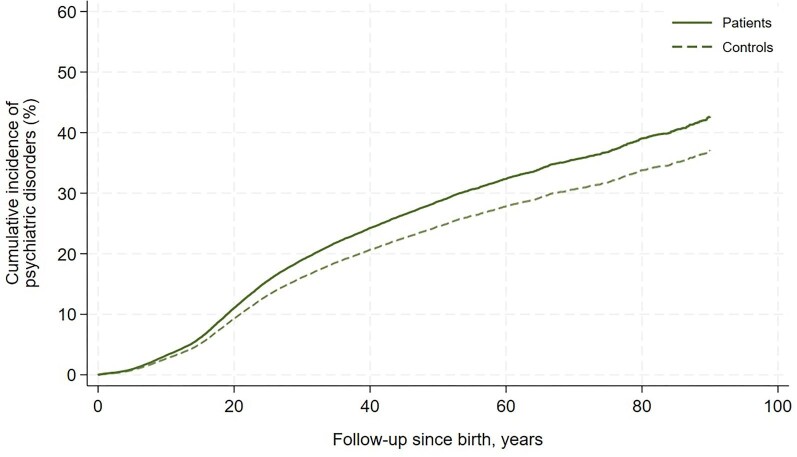
Cumulative incidence of psychiatric disorders with death as competing risk in patients with an isolated congenital ventricular septal defect and matched controls from the general Danish population.

Overall, psychiatric morbidity and use of psychotropic agents were more common in patients with VSD than controls from the general Danish population, presented in *Table 2*. The most pronounced difference between patients and controls within psychiatric disorders was found within the category intellectual disabilities. *Figure 2* illustrates the cumulative incidence of psychiatric disorders in VSD patients compared with controls. The cumulative incidence increased at a higher rate in the patients relative to the controls during early adulthood but waned around the fifth decade of life where the rates remained almost parallel. At the age of 40 years, the unadjusted rates of psychiatric disorders were 24% (95% CI: 23–25%) and 21% (95% CI: 20–21%) in patients with VSD and controls, respectively (*P* < 0.001). There was no difference between sex in developing a psychiatric disorder [HR 1.06 (95% CI: 0.95–1.18)]. The cumulative incidence of prescription of psychotropic agents is graphically illustrated in Supplementary material online, *Figure S2*.

**Table 2:** Psychiatric disorders and use of psychotropic agents

	Number of event/total number of individuals (%)		Incidence rate per 10 000 Person-years		
	VSD	Controls	VSD	Controls	HR (95% CI)
Burden of psychiatric disorders					
All psychiatric disorders (F10–98)	1261 (15.8)	10 727 (13.5)	64.3	52.2	1.24 (1.17–1.32)
Mental and behavioural disorders due to psychoactive substance use (F10–19)	281 (3.5)	2651 (3.3)	18.9	17.0	1.13 (1.00–1.29)
Schizophrenia or psychosis (F20–29)	96 (1.2)	819 (1.0)	6.3	4.8	1.31 (1.06–1.63)
Mood affective disorders (F30–39)	260 (3.2)	2588 (3.3)	16.8	14.4	1.20 (1.05–1.37)
Emotional disorders (F40–48)	516 (6.4)	4252 (5.3)	33.8	26.5	1.29 (1.17–1.42)
Behavioural syndromes associated with physiological disturbances and physical factors (F50–59)	71 (0.9)	720 (0.9)	4.9	4.7	1.03 (0.81–1.31)
Personality and behavioural disorders (F60–69)	133 (1.7)	1162 (1.5)	8.4	7.0	1.21 (1.00–1.45)
Intellectual disabilities (F70–79)	144 (1.8)	397 (0.5)	8.5	2.2	3.66 (2.98–4.50)
Developmental disorders (F80–89)	162 (2.0)	1199 (1.5)	10.1	7.4	1.33 (1.12–1.58)
Behavioural and emotional disorders (F90–98)*	263 (3.3)	2330 (2.9)	17.2	14.7	1.15 (1.01–1.31)
Use of psychotropic agents					
All prescribed medicine	1935 (24.1)	18 321 (23.0)	133.0	119.0	1.14 (1.09–1.20)
Antipsychotics	1120 (14.0)	10 562 (13.3)	23.5	20.7	1.15 (1.08–1.22)
Antidepressants	481 (6.0)	4383 (5.5)	56.4	51.2	1.18 (1.08–1.30)
Anxiolytics	828 (10.3)	7325 (9.2)	41.5	35.3	1.23 (1.15–1.33)
Hypnotics	831 (10.4)	7892 (9.9)	41.1	37.8	1.15 (1.07–1.23)
Psychostimulants	248 (3.1)	2346 (2.9)	12.0	11.0	1.08 (0.95–1.24)

Data are presented as absolute numbers with percentage and HR with 95% confidence interval computed using Cox proportional hazards regression model where patients with isolated VSD were compared with matched controls from the general Danish population. HR, hazard ratio; VSD, ventricular septal defect.

*Onset in childhood and adolescence

### Education and work participation

**Figure 3:**
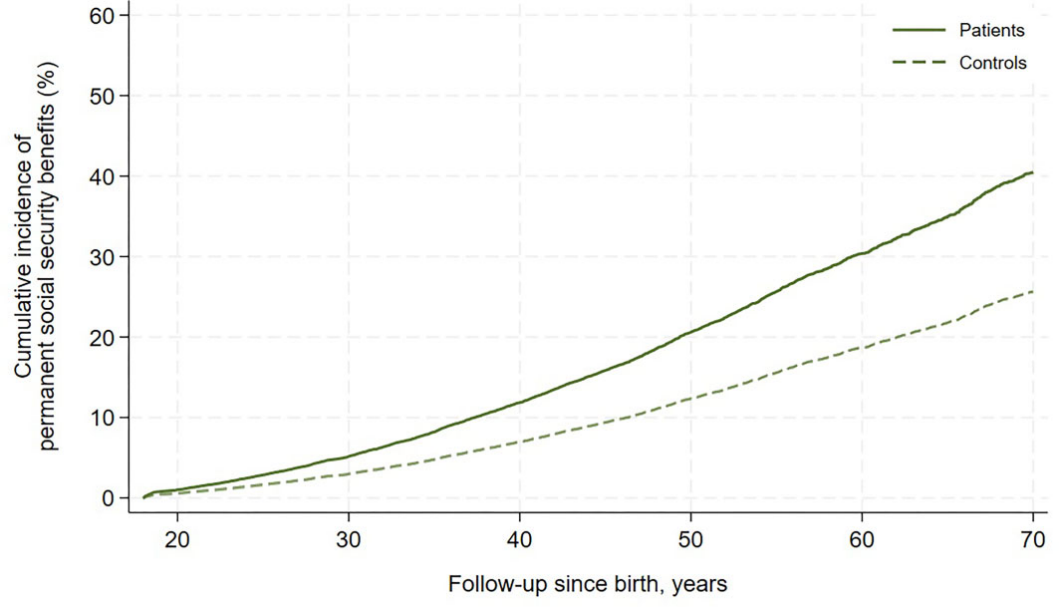
Cumulative incidence of retrieval of permanent social security benefits with death as competing risk in patients with an isolated congenital ventricular septal defect and matched controls from the general Danish population.

**Figure 4:**
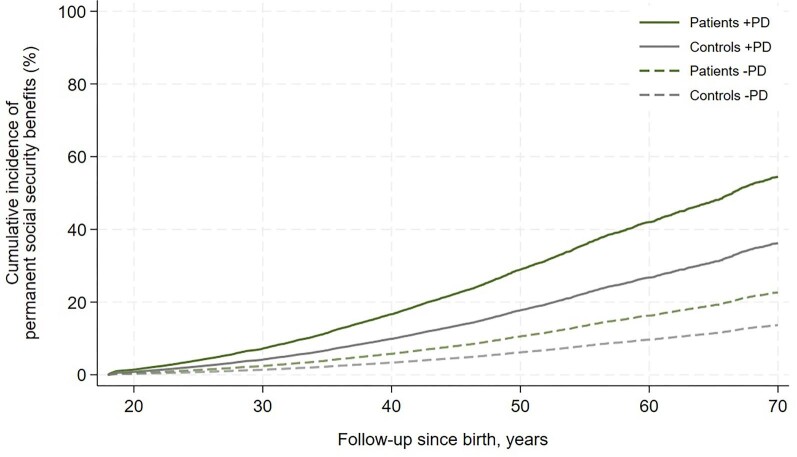
Cumulative incidence of retrieval of permanent social security benefits stratified by presence of psychiatric disorder and with death as competing risk in patients with an isolated congenital ventricular septal defect and matched controls from the general Danish population. PD, psychiatric disorder.

Data on education was available in approximately half of the cohort. Patients with VSD generally had a lower level of education than the controls, presented in *Table 1*. A larger proportion of VSD patients had a basic education as the highest level of education as compared with the controls, and conversely a larger proportion of the controls had a higher education as the highest level of education, driven by medium and long cycle education representing three or more years at the university. Data on work participation was available in 60% and 63% of the patient and control cohort, respectively. Patients with VSD had a lower work participation than the controls explained by an increased proportion of time as unemployed, on long-term sick leave, or on permanent social security benefits, presented in *Table 3*. Isolating the analysis to patients and controls with a psychiatric disorder, the lower work participation in VSD patients persisted but was solely driven by increased proportion of time on permanent social security benefits. The time-to-event analysis of receiving permanent social security benefits in patients with VSD compared with controls revealed a HR of 1.77 (95% CI: 1.62–1.93) in the total cohort, a HR of 1.73 (95% CI: 1.53–1.97) in the cohort without psychiatric disorders, and a HR of 1.54 (95% CI: 1.34–1.77) in the cohort with psychiatric disorders. The cumulative incidence of receiving permanent social security benefits for the total cohort is graphically illustrated in *Figure 3*. VSD patients with a psychiatric disorder displayed a HR of 3.20 (95% CI: 2.70–3.79) compared with patients without a psychiatric disorder. Similar comparison in the control cohort yielded a HR of 3.80 (95% CI: 3.55–4.06). The corresponding cumulative incidence of the latter subanalyses are graphically illustrated in *Figure 4*. The rates of receiving permanent social security benefits at 40 years of age as VSD patient with psychiatric disorder was 29% (95% CI: 25–33%) and 8% (95% CI: 7–9%) without psychiatric disorder. The corresponding rates for the controls were 21% (95% CI: 19–22%) and 4% (95% CI: 4–4%). Cumulative incidence using Kaplan–Meier failure function with patients at risk is presented in Supplementary material online, *Figure S3*. No correlation between individual psychiatric disorder and work participation could be demonstrated, as presented in Supplementary material online, *Table S5*.

**Table 3:** Work participation

	Total cohort			Psychiatric disorders		
	Patients (*n* = 4764)	Controls (*n* = 50 259)	*P*-value	Patients (*n* = 1067)	Controls (*n* = 9311)	*P*-value
Working	83.8%	86.9%	<0.0001	71.5%	74.5%	0.0018
Unemployment	9.7%	7.9%	<0.0001	18.1%	17.6%	0.5330
Long-term sick leave	2.0%	1.2%	<0.0001	1.9%	2.0%	0.8358
Permanent social security benefits	8.1%	4.3%	<0.0001	15.9%	10.2%	<0.0001

Data are presented as the proportion of time working, unemployed, on long-term sick leave, or permanent social security benefits in the total cohort of patients with isolated congenital ventricular septal defect and their matched controls from the general Danish population and of the subcohort of patients and matched controls with psychiatric disorders.

## Discussion

This Danish nationwide cohort study of 8006 patients with an isolated congenital VSD revealed an increased burden of psychiatric morbidity, higher use of psychotropic agents, lower level of education, and lower work participation compared with controls from the general population. Notably, patients and controls with known chromosomal abnormalities were excluded. While our study adds new knowledge regarding the long-term outcome of patients with congenital VSD, it is one of several recently published studies that highlight the not-so-benign outcome of patients with simple congenital cardiac defects. Patients with congenital VSD have increased mortality^12,19,20^ and more morbidity^13,20–23^ compared with the general population, and this study is the first to reveal a high burden of psychiatric disorders and issues with education and work participation. Four findings need to be highlighted. First, patients with VSD displayed a higher cumulative incidence of psychiatric disorders than the controls. Second, at 40 years of age, 13% of patients with VSD received permanent social security benefits compared with 7% of controls. Third, both patients and controls with psychiatric disorders were more likely to retrieve permanent social security benefits than those without, but patients with psychiatric disorders were also more likely to retrieve permanent social security benefits than controls with psychiatric disorders. Fourth, the proportion of VSD patients fulfilling a higher level of education was smaller than the proportion of controls. Importantly, these findings emphasize the necessity for a broader perspective in the follow-up of these patients, not only focusing on the cardiovascular system, but also uncover mental health and work-related issues.

We believe we are the first to systematically demonstrate an increased burden of psychiatric disorders in patients with isolated congenital VSD on a populational level. Nyboe *et al*. previously demonstrated an increased risk of psychiatric morbidity in patients with isolated ASD,^9^ but we can only speculate on whether these two simple shunt-lesions share the same pathophysiological mechanism. A two-fold increased risk of psychiatric disorders was furthermore identified in a Finish nationwide cohort of 1428 patients with VSDs surgically closed in early childhood^21^ and an increased risk of psychiatric disorders was found in 6927 patients with all types of congenital heart diseases regardless of adjusting for age, sex, chromosomal abnormalities, parental psychiatric status, and intervention (surgery or percutaneous transcatheter procedure).^24^

Dojvak *et al*. investigated the burden of extracardiac anomalies in foetuses with congenital heart disease by conducting foetal magnetic resonance imaging.^14^ Across the spectrum of congenital heart diseases, foetuses with VSD and ASD presented the highest prevalence of extracardiac anomalies (74%), predominantly related to the central nervous system (43%). More specifically, foetuses with VSD or ASD were those where developmental anomalies in the midbrain and hindbrain (21%), ventral prosencephalon (7%) , and dorsal prosencephalon (26%) were most commonly detected.^14^ Abnormal cerebrospinal fluid space was seen in approximately one fifth of the foetuses with VSD or ASD. The high prevalence of structural brain anomalies is striking with regards to the anatomical simplicity of the defects. Genetics likely play a role as shared genetic contributions to congenital heart disease, extra-cardiac anomalies, and neurodevelopmental disabilities have been described.^25^ In a recent publication in Science, Zhao *et al*. demonstrated causal genetic effects between cardiac dysfunction and brain disorders and hinting at potential connections between cardiovascular disease and neurological health.^26^ Altogether, it might not be the VSD per se driving the psychiatric disorder, but rather an underlying disturbance in the neurodevelopment.

Asschenfeldt *et al*. demonstrated an association between abnormal left-hemispheric sulcal folding patterns and neurodevelopmental challenges in terms of intelligence, executive function, and visuospatial abilities in adult patients with an isolated and surgically closed VSD.^27^ Furthermore, an Austrian retrospective cohort study of more than 200 children with congenital heart disease demonstrated that 29% of patients with VSD were in need for special schooling; the highest prevalence across all congenital heart morphologies^28^ and a Danish population-based and nationwide cohort study revealed that 18% of patients with simple and 24% of patients with complex congenital heart disease were in need for special schooling (compared with 10% in subjects without congenital heart disease) with the difference evident already from first grade.^29^ Our data on lower level of education and higher risk for intellectual disabilities in patients with VSD compared with the general population are supportive in this regard. While it is clear that patients with simple lesions such as the VSD suffer not only from cardiovascular morbidity, but also psychiatric morbidity the aetiology remains hitherto unknown but probably encompass a complex interaction between biological and socioenvironmental factors. Lower blood oxygen tension has been postulated to be the explanation to the observed abnormal brain development in patients with complex congenital heart diseases.^30,31^ Even though the VSD represents left-to-right shunt and is an acyanotic condition with expected normal systemic oxygenation, patients with VSD are associated with significant congestive heart failure and a particularly failure to thrive during childhood. Consequently, patients fall off the growth curve during the first year of life and it is conceivable to hypothesize a negative impact also on brain development. The fact that patients with surgically closed VSD were not at higher risk of psychiatric disorders in combination with the increased risk of psychiatric disorders in the total cohort of patients with acyanotic isolated VSDs indicates that other aetiologies than cerebral embolization during intervention, reduced cerebral blood flow during cardiopulmonary bypass, and chronic cerebral hypoxia are at play.

Lau-Jensen *et al*. demonstrated an increased burden of attention deficit/hyperactivity disorder in adult patients with surgically or percutaneously closed ASD or VSD compared with matched controls.^32^ Furthermore, El Dabagh *et al*. demonstrated dysfunctional cognitive abilities,^16^ namely executive functions, which is associated with emotional distress^33^ and lower HRQoL^34^ in young adult patients with surgically closed congenital ASD or VSD and in a large study, including 1478 patients from the US, patients with mild congenital heart diseases, such as shunts, were significantly more likely to have cognition disabilities than the general population.^35^ In a recent published paper by Maagaard *et al*. on self-assessed quality of life in patients with isolated congenital VSD, patients reported lower self-perceived physical functioning, reduced general health perception as well as higher levels of stress as compared with matched controls.^36^ Interestingly, these issues, namely perceived health complaints and limitations in daily physical activities, are associated with unemployment, fewer working hours, and limitations at work^37^ which in turn can be driven by chronic diseases.^38^ Given the fact that patients with VSD display lower level of education and to a larger extent suffer from psychiatric and chronic somatic diseases^13^ compared with their peers, the lower work participation might not come as a surprise.

The overall unemployment rate in patients with unselected congenital heart diseases has been reported from 24% to 33%.^3,37,39^ In the current study, patients with VSD displayed lower work participation with increased proportions of unemployment, long-term sick leave, and permanent social security benefits compared with controls from the general population. The difference between patients and controls in receiving permanent social security benefits were evident already at 20 years of age and increased continuously throughout life. The distinct difference already in early adulthood might indicate inherited issues resulting in lower levels of education and psychiatric morbidity since somatic morbidity is trivial prior to the fourth decade of life in patients with VSD.^13^ Our data revealed a three-fold increased risk of receiving permanent social security benefits if diagnosed with a psychiatric disorder compared with no psychiatric disorder, regardless of presence of a VSD. On the other hand, absence of psychiatric disorders still yielded an almost two-fold increased risk in VSD patients compared with controls. Thus, lower work participation is only partly, not completely, driven by psychiatric morbidity.

Irrespective the underlying cause of the increased burden of psychiatric morbidity, the lower level of education, and the lower work participation, these areas warrant early recognition in the routine follow-up already in childhood and adolescence and importantly a cautious and vigilant transition from paediatric to adult care for at-risk-patients is paramount.

### Limitations

The DREAM database include data from 1991. Subjects <18 years of age were excluded in the analysis (*n* = 30 536) and we lack data on patients and controls who deceased prior to the introduction of the DREAM database (*n* = 204 and *n* = 241, respectively). Therefore, data on work participation was only available for approximately two-thirds of the cohort. We do not know if the latter group of patients used social services differently or had an alternative association with work force than those who entered the system. The survival of patients from the historic era (before 1990) was lower compared with patients from the modern era^12^ and patients from the historic era could be expected to have an increased burden of morbidity, we therefore believe the potential use of social security benefits would be at least the same, or higher, as those alive entering the database. These circumstances would generate an even stronger association.

The validity and accuracy of our estimates depends on correct coding of the VSD and psychiatric diagnoses. The positive predictive value of VSD diagnosed at university hospitals is high in DNPR^40^ as is the predictive value of psychiatric disorders in the Danish Central Psychiatry Registry.^41^ Data on out-patient contacts for psychiatric disorders was unfortunately only available after 1995 in the Danish Central Psychiatry Registry. We do not expect any difference between out-patient and in-hospital diagnoses in patients and controls. However, bias might arise if VSD patients are referred to psychiatric departments for clinical assessment and subsequently being diagnosed with a psychiatric disorder more frequently than their peers which we cannot disregard and would generate an overestimation of the estimates. On the other hand, it is possible that psychiatric disorders might be easily disregarded in patients with chronic somatic disease, potentially leading to an underestimation of the findings.

The nationwide registries do not include clinical data. While the absence of clinical data is not inherently a limitation, it does impede the ability to finely categorize patient groups and restricts our ability to gain in-depth physiological insights into potential causes. For example, because we lacked shunt data, we could not categorize unrepaired defects as small and restrictive; instead, we could only speculate that these defects remained unrepaired due to their perceived nature. Additionally, patients with spontaneously closed VSD are likely to be included in the study. Whether these patients demonstrate a similar pattern in terms of psychiatric morbidity and work participation is unknown, but nonetheless a thought-provoking area for future research.

Data on socioeconomic status and parental psychiatric status were not available restricting us from adjusting psychiatric morbidity for these factors. However, previous studies on patients with ASD and the population of patients with congenital heart disease as a whole have demonstrated that psychiatric morbidity is not driven by these factors.^9,24^

Neurodevelopmental disturbances might explain the significant proportion of patients with intellectual disabilities, but could also partially or entirely be driven by undiagnosed chromosomal abnormalities why these estimates should be interpreted with caution.

## Conclusion

This nationwide cohort study of 8006 patients with isolated congenital VSD revealed significant extracardiac long-term issues with increased burden of psychiatric disorders, lower level of education, and lower work participation compared with controls from the general population. Our data, combined with what has previously been published, suggest that it is not the VSD itself, with its potential haemodynamic complications, causing the findings, but are most likely due to associated, yet undiagnosed, extracardiac lesions and neurodevelopment disturbances. Furthermore, our data emphasizes the significance of taking a broader perspective in the longitudinal follow-up of children and adults with congenital heart diseases. It is crucial to focus not only on cardiovascular morbidity but also on mental health, education, and subsequent employment as these factors severely affect quality of life and have direct socioeconomic implications on an individual and societal level.

## Supplementary Material

qcad072_Supplemental_File

## Data availability

The data underlying this article cannot be shared publicly in accordance with the directive from the central authority on Danish Statistics, Statistics Denmark.
