# Supplementary material for: Psychiatric morbidity and work participation in patients with congenital ventricular septal defects: a case-controlled study
Source: Eur Heart J Qual Care Clin Outcomes. 2024 Jan 4;10(6):552–61. doi: 10.1093/ehjqcco/qcad072 (PMC11398907; doi:10.1093/ehjqcco/qcad072)
Supplement: qcad072_Supplemental_File [file qcad072_supplemental_file.docx]

Supplemental Material

**Table S1.** Codes according to the International Classification of Disease eighth and tenth edition used to identify concomitant congenital cardiac malformations.

| Malformation | ICD-8 | ICD-10 |
| --- | --- | --- |
| Truncus communis | 74609 | Q200 |
| Transposition of the great arteries | 74619 | Q203, Q203A |
| Congenitally corrected transposition of the great arteries |  | Q205, Q205A, Q205B |
| Univentricular heart (DOLV, DILV and DIRV) |  | Q202, Q204 |
| Atrial septal defect | 74640, 74641, 74649 | Q211, Q211A, Q211B |
| Atrioventricular septal defect | 74659 | Q212, Q218B |
| Tetralogy of Fallot | 74629 | Q201, Q213, Q213A |
| Aortopulmonal septal defect |  | Q214 |
| Atresia of the pulmonary artery | 74739 | Q220, Q255 |
| Pulmonary stenosis and insufficiency | 74663 | Q221, Q222, Q223, Q243, Q256 |
| Atresia or stenosis of the tricuspid valve, hypoplastic right heart | 74661 | Q224, Q226 |
| Hypoplastic left heart syndrome |  | Q234 |
| Aortic stenosis and insufficiency | 74662 | Q230, Q231, Q253, Q244 |
| Mitral valve disease | 74660 | Q232, Q233 |
| Cor triatriatum, coronary anomaly, congenital heart block |  | Q242, Q245, Q246 |
| Patent ductus arteriosus* | 74709 | Q250 |
| Coarctation of the aorta | 74719 | Q251, Q251A |
| Other malformations of the aorta (including aortic atresia) | 74729 | Q254, Q238, Q252 |
| Other malformations of the pulmonary artery |  | Q257, Q258 |
| Partial anomalous pulmonary venous drainage |  | Q263 |
| Total anomalous pulmonary venous drainage |  | Q262 |

DILV, double-inlet left ventricle; DIRV, double-inlet right ventricle; DORV, double-outlet right ventricle; ICD, international classification of disease. *Surgically closed

## **Table S2**. Codes according to the International Classification of Disease eighth and tenth edition used to identify surgical or transcatheter closure of defect.

| Intervention | ICD-8 | ICD-10 |
| --- | --- | --- |
| Surgery | 30801^†^, 30809^†^, 30819^†^, 30899^†^, 31460^‡^, 31540^‡^ | KFHB00, KFHB10, KFHB20, KFHB30, KFHB40, KFHB50, KFHB60, KFHB70, KFHB80, KFHB96, KFHC00, KFHC10, KFHC20, KFHC30, KFHC96 |
| Percutaneous transcatheter | - | KFHB42 |

ICD, International Classification of Disease.

^†^Codes used during the period 1989-1995.

^‡^Codes used during the period 1973-1988.

**Table S3.** Codes according to the International Classification of Disease eighth and tenth edition used to identify psychiatric disorders.

| Psychiatric morbidity | ICD-8 | ICD-10 |
| --- | --- | --- |
| F10-19 | 29129, 29139, 29199, 29430, 29409, 29109, 29309, 29319, 29320, 29329, 29328, 29429, 29438, 29439, 29449, 29469, 29479, 29489, 29499 | DF10*, DF12*, DF13*, DF14*, DF15*, DF16*, DF17*, DF18*, DF19* |
| F20-29 | 29509, 29519, 29529, 29539, 29549, 29559, 29569, 29579, 29589, 29599, 29709, 29719, 29799 | DF20*, DF21*, DF22*, DF23*, DF24*, DF25*, DF28*, DF29* |
| F30-39 | 29609, 29619, 29629, 29699, 29689, 29699, 29809, 29819 | DF30*, DF31*, DF32*, DF33*, DF34*, DF38*, DF39* |
| F40-49 | 30009, 30019, 30029, 30039, 30049, 30059, 30069, 30079, 30089 | DF40*, DF41*, DF42*, DF43*, DF44*, DF45*, DF48* |
| F50-59 | 30649, 30560, 30568, 30569, 30650, 30658, 30659, 30999 | DF50*, DF51*, DF52*, DF53*, DF54*, DF55*, DF59* |
| F60-69 | 29900, 29901, 29902, 29903, 29904, 29905, 29909, 30109, 30119, 30129, 30139, 30149, 30159, 30169, 30179, 30180, 30181, 30182, 30183, 30184, 30189, 30199, 30229, 30289, 30299 | DF60*, DF61*, DF62*, DF63*, DF65*, DF66*, DF68*, DF69* |
| F70-79 | 31090, 31099, 31190, 31199, 31290, 31299, 31390, 31399, 31590, 31599, 31490, 31499 | DF70*, DF71*, DF72*, DF73*, DF78*, DF79* |
| F80-89 | 30610, 30611, 30612, 30618, 30619, 30629, 30639, 30649, 30800, 30801, 30802, 30803, 30804, 30805, 30806, 30807, 30809 | DF80*, DF81*, DF82*, DF83*, DF84*, DF88*, DF89* |
| F90-99 | - | DF90*, DF91*, DF92*, DF93*, DF94*, DF95*, DF98* |

*Include all lower level of diagnose codes.

**Table S4.** Codes according to the Anatomical Therapeutic Chemical classification used to identify prescription of psychotropic agents.

| Drug | Anatomical Therapeutic Chemical code |
| --- | --- |
| Antipsychotics | N05A*, N03AF01 |
| Antidepressants | N06A* |
| Anxiolytics | N05B* |
| Hypnotics | N05C* |
| Psychostimulants | N06BA04, N06BA09 |

*Include all lower level of diagnose codes.


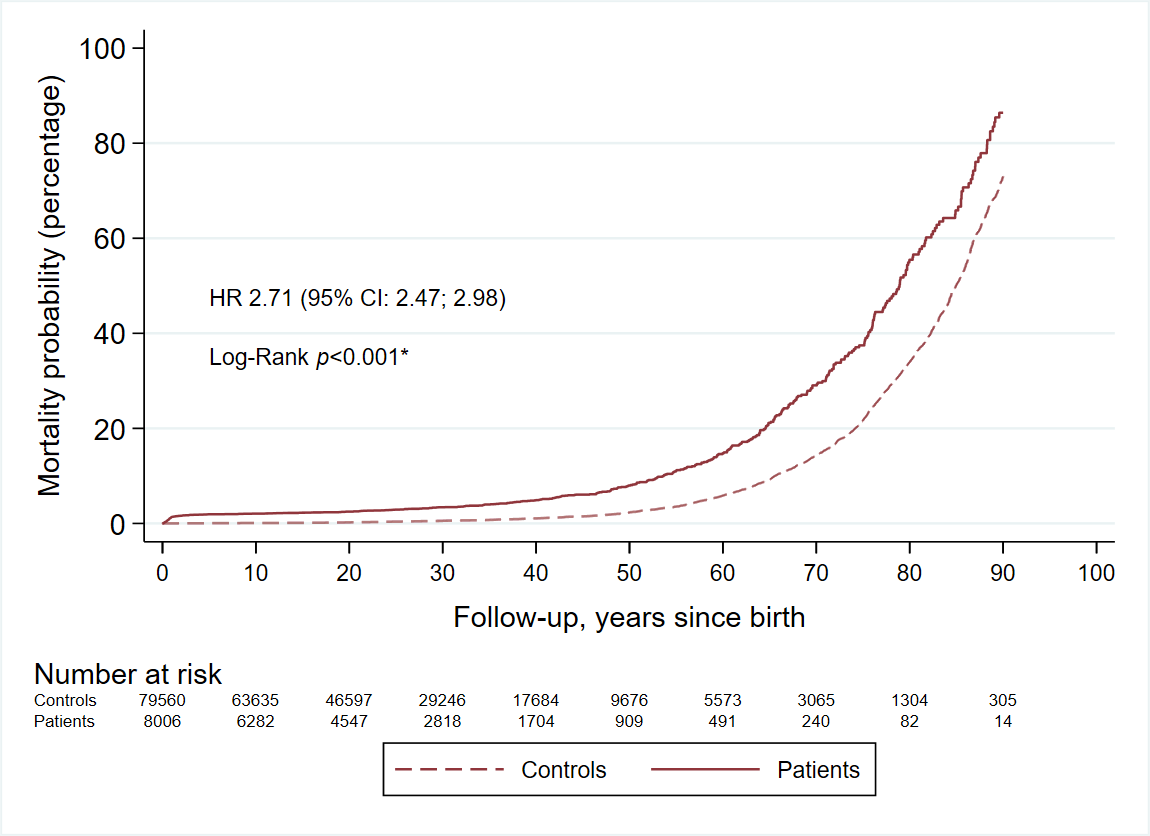
**Figure S1.** Cumulative mortality in patients with isolated congenital ventricular septal defect and matched controls from the general Danish population.

CI, confidence interval; HR, hazard ratio.

**Figure S2.** Cumulative incidence of the use of psychotropic agents with death as competing risk in patients with isolated congenital ventricular septal defect and matched controls from the general Danish population.


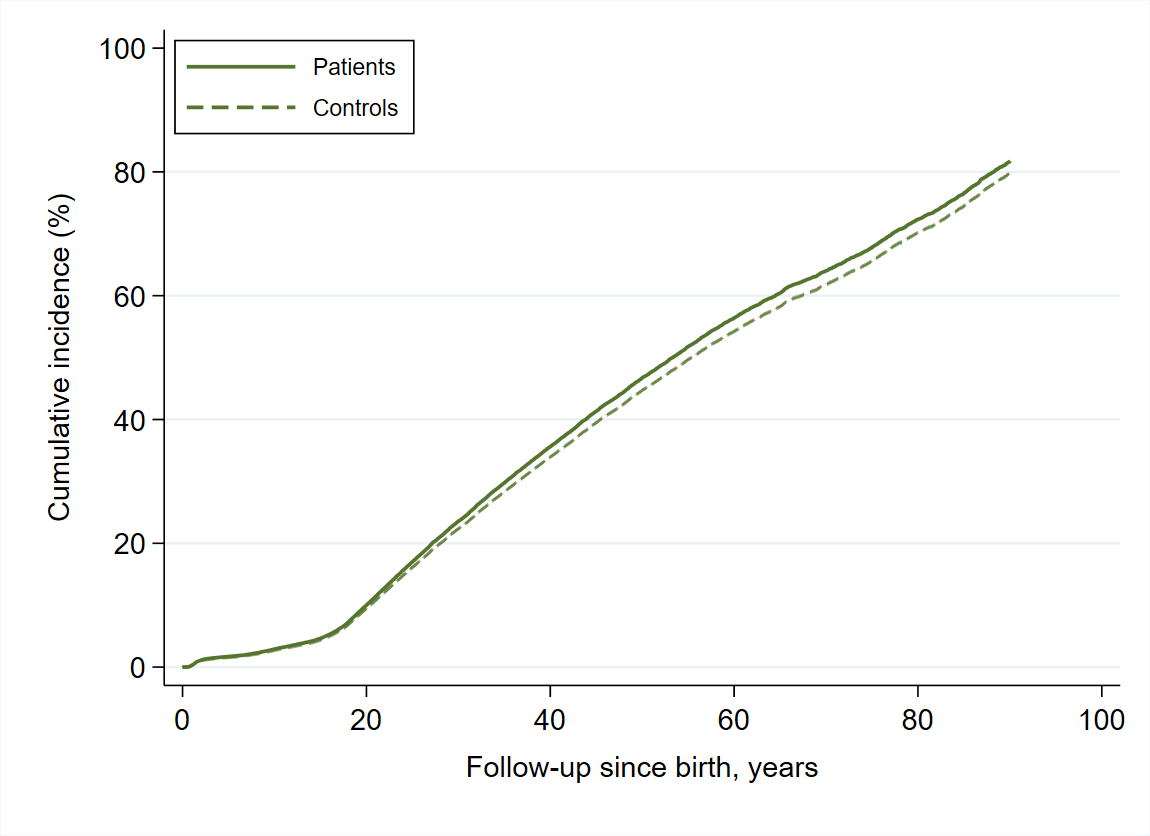


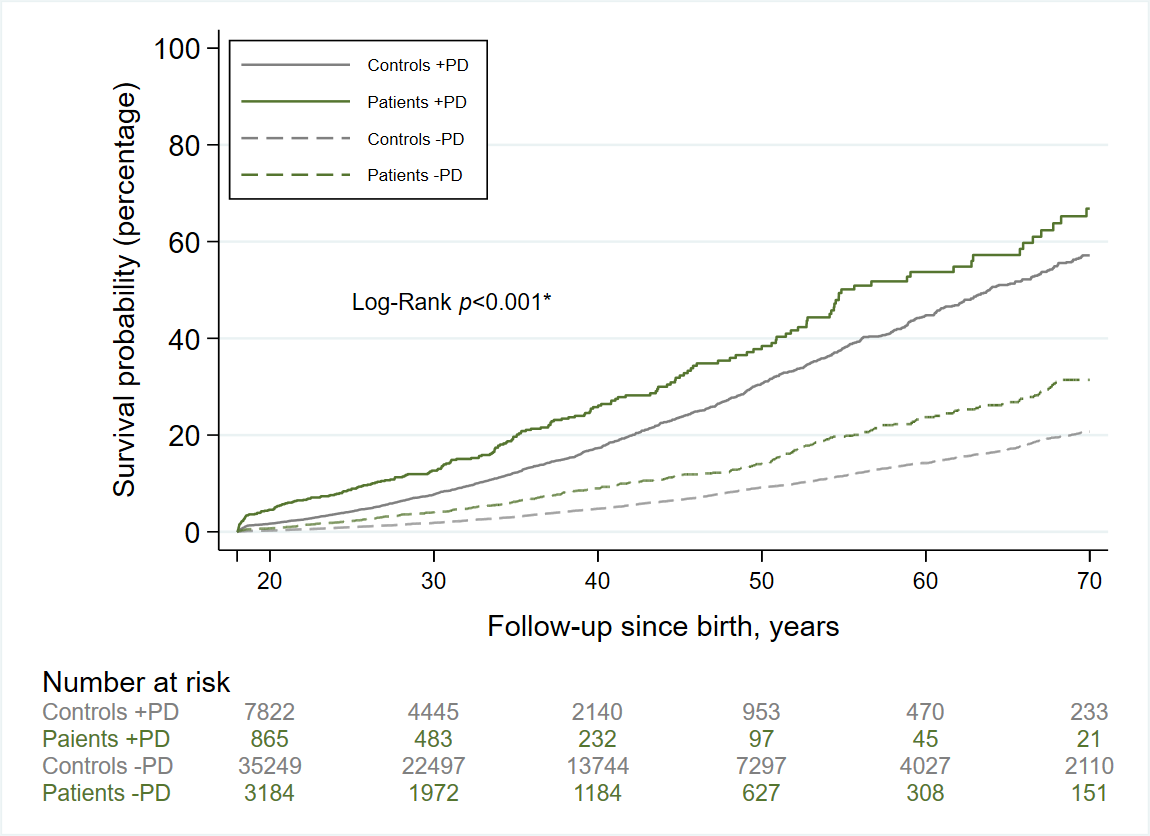
**Figure S3.** Kaplan-Meier cumulative incidence of use of permanent social security benefits stratified by psychiatric disorders in patients with isolated congenital ventricular septal defects and matched controls from the general Danish population.

PD, psychiatric disorder.

**Table S5.** Permanent social security benefits and psychiatric disorders.

|  | Permanent social security benefits | |
| --- | --- | --- |
| Psychiatric disorder | **Not Receiving/Total Number of Individuals (%)** | **Receiving/Total Number of Individuals (%)** |
| Mental and behavioral disorders due to psychoactive substance use (F10-19) | 131 (84) | 25 (16) |
| Schizophrenia or psychosis (F20-29) | 4 (36) | 7 (64) |
| Mood affective disorders (F30-39) | 76 (79) | 20 (21) |
| Emotional disorders (F40-48) | 251 (79) | 66 (21) |
| Behavioral syndromes associated with physiological disturbances and physical factors (F50-59) | 49 (91) | 5 (9) |
| Personality and behavioral disorders (F60-69) | 74 (70) | 32 (30) |
| Intellectual disabilities (F70-79) | 16 (21) | 60 (79) |
| Developmental disorders (F80-89) | 49 (71) | 20 (29) |
| Behavioral and emotional disorders (F90-98) | 145 (80) | 37 (20) |

Proportions of individuals receiving permanent social security benefits stratified by specific psychiatric disorder in the population of patients with VSD. Data are reported as absolute numbers with percentages. Patients only contribute to one category of psychiatric disorder, namely the one that was diagnosed first. Consequently, the numbers within each category differ compared to Table 2.
